# Combination therapy of an ^211^At-labeled RGD peptide and immune checkpoint blockade to enhance antitumor efficacy

**DOI:** 10.1007/s00259-025-07498-3

**Published:** 2025-08-06

**Authors:** Hiroaki Echigo, Masayuki Munekane, Takeshi Fuchigami, Kohshin Washiyama, Takashi Nakamura, Atsushi Furukawa, Zhuoqing Chen, Kenji Mishiro, Hiroshi Wakabayashi, Kazuhiro Takahashi, Seigo Kinuya, Kazuma Ogawa

**Affiliations:** 1 Graduate School of Medical Sciences, Kanazawa University, Kakuma-machi, Kanazawa, 920-1192 Ishikawa Japan; 2 Advanced Clinical Research Center, Fukushima Global Medical Science Center, Fukushima Medical University, 1 Hikarigaoka, Fukushima, 960-1295 Japan; 3 Department of Nuclear Medicine, Kanazawa University Hospital, Kanazawa University, Takara-machi 13-1, Kanazawa, 920-8641 Ishikawa Japan

**Keywords:** Astatine-211, Targeted alpha therapy, RGD peptide, Immune checkpoint blockade, Combination therapy

## Abstract

**Purpose:**

Recently, an ^211^At-labeled RGD peptide with an albumin-binding moiety, Ga-DOTA-K([^211^At]APBA)-c(RGDfK) ([^211^At]**1**), was developed for targeted alpha therapy and radiotheranostics. [^211^At]**1** showed high tumor accumulation and inhibited tumor growth in a dose-dependent manner. This study aimed to investigate whether [^211^At]**1** can induce an antitumor immune response and whether the combination of [^211^At]**1** and immune checkpoint blockade can enhance therapeutic efficacy.

**Methods:**

Biodistribution experiments of [^211^At]**1** and therapeutic experiments of [^211^At]**1** with or without anti-PD-1 or anti-CTLA-4 antibody were conducted in Colon-26 tumor-bearing BALB/c mice. Additionally, therapeutic experiments of [^211^At]**1** were conducted in Colon-26 tumor-bearing BALB/c nu/nu mice to confirm the difference in the therapeutic effects derived from antitumor immune responses of [^211^At]**1**. Infiltration of antitumor immune activity in Colon-26 tumors was confirmed by flow cytometry and immunofluorescence staining.

**Results:**

[^211^At]**1** showed high tumor accumulation and inhibited tumor growth in a dose-dependent manner in Colon-26 tumor-bearing BALB/c mice. Additionally, mice treated with [^211^At]**1** (675 kBq) and anti-CTLA-4 antibody showed superior tumor growth inhibition compared with mice treated with only [^211^At]**1** (675 kBq). Tumor growth inhibition was weaker in BALB/c nu/nu mice than in BALB/c mice. Infiltration of CD4^+^ and CD8^+^ T cells was observed following treatment with [^211^At]**1** (675 kBq) alone or in combination with anti-CTLA-4 antibody.

**Conclusion:**

[^211^At]**1** induced an antitumor immune response and enhanced the therapeutic efficacy. The combination of [^211^At]**1** and immune checkpoint blockade agents could be promising for cancer therapy.

**Supplementary Information:**

The online version contains supplementary material available at 10.1007/s00259-025-07498-3.

## Introduction

Immune checkpoint blockade (ICB) therapy has gathered much attention in cancer treatment due to its effectiveness [1]. ICBs inhibit cytotoxic T-lymphocyte antigen 4 (CTLA-4), programmed death 1 (PD-1), and PD-1 ligand 1 (PD-L1), which inhibit antitumor immune responses in the tumor microenvironment, and promote antitumor efficacy [2]. However, the overall response rate to a single ICB therapy is only approximately 20% [3]. Recently, several studies have revealed that inducing antitumor immune responses via other therapies, such as radiotherapy, can enhance the therapeutic efficacy of ICB [1, 3–6]. Radiotherapy destroys tumors by inducing DNA damage in cancer cells through direct action or the production of reactive oxygen species [3, 7]. In the process of dying by radiotherapy, cancer cells release damage-associated molecular patterns (DAMPs), such as adenosine triphosphate, high-mobility group box 1 protein, and calreticulin, causing immunogenic cell death (ICD) [3, 4, 7, 8]. Additionally, radiotherapy stimulates the secretion of cytokines and chemokines, promotes the maturation of dendritic cells and activation CD8^+^ T cells, and induces antitumor immune responses [3, 4, 7, 8]. Therefore, the combing radiotherapy with ICB therapy could increase its therapeutic efficacy and is widely used in clinical [9–11].

Targeted radionuclide therapy (TRT) has been reported to induce antitumor immune responses similar to those induced by radiotherapy and increases the expression of PD-1 and CTLA-4 in cytotoxic T cells and PD-L1 in surviving cancer cells [12]. Therefore, several studies have shown that TRT and ICB combination therapy could enhance the therapeutic effects [13, 14]. Among TRT, targeted alpha therapy (TAT) has attracted much attention due to its stellar therapeutic effects [15, 16]. Previous studies have shown that combination therapy of TAT and immunotherapy enhances the therapeutic efficacy [17–19]. Although the mechanisms underlying TAT-induced antitumor immune responses were unknown, Ertveldt et al. reported that the mechanisms underlying TAT-induced antitumor immune responses were similar to those of radiotherapy and other TRT [20].

**Fig. 1:**
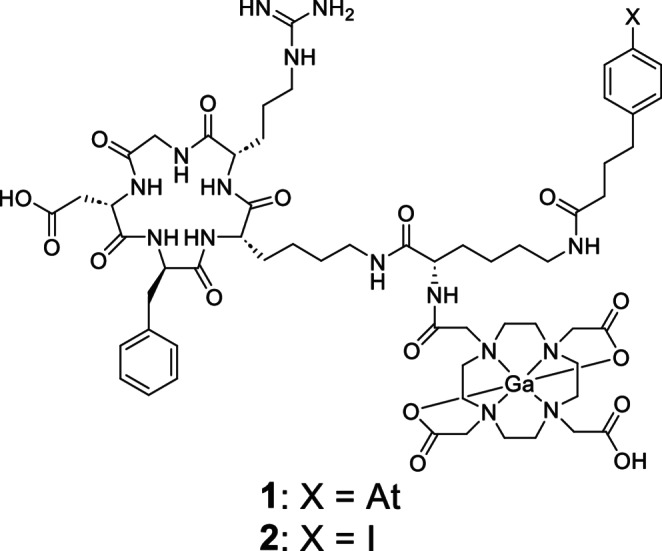
Chemical structures of Ga-DOTA-K(APBA)-c(RGDfK) (**1**) and Ga-DOTA-K(IPBA)-c(RGDfK) (**2**)

We previously developed ^211^At-labeled Arg-Gly-Asp (RGD) peptide derivatives for TAT and radiotheranostics [21–24]. Among them, Ga-DOTA-K([^211^At]APBA)-c(RGDfK) ([^211^At]**1**, Fig. 1) with 4-(4-astatophenyl)butyric acid (APBA) as albumin-binding moiety showed high tumor accumulation and inhibited tumor growth in a dose-dependent manner [24]. However, the therapeutic effects of [^211^At]**1** were not enough because the tumor was hardly reduced and regrew after days. Therefore, this study aimed to investigate whether [^211^At]**1**, a TAT agent, can induce an antitumor immune response and whether the combination of [^211^At]**1** and ICB can enhance therapeutic efficacy. In our previous studies, we evaluated radiolabeled RGD peptides in a tumor-bearing BALB/c nu/nu mouse model inoculated with U-87 MG human glioblastoma cells that highly expressed α_v_β_3_ integrin as a target of RGD peptides. In this study, a tumor-bearing BALB/c mouse model inoculated with Colon-26 mouse colon cancer cells, which are derived from the same species and are frequently used in basic cancer research [25, 26], was used to evaluate the antitumor immune responses after [^211^At]**1** treatment.

## Materials and methods

### General

^211^At was produced on CYPRIS MP-30 cyclotron (Sumitomo Heavy Industries, Ltd., Tokyo, Japan) in the Advanced Clinical Research Center at Fukushima Medical University [27]. Colon-26 cells were obtained from Cell Resource Centre for Biomedical Research in Tohoku University (Sendai, Japan). InVivoMAb anti-mouse PD-1 (CD279) (αPD-1) and InVivoMAb anti-mouse CTLA-4 (CD152) (αCTLA-4) were purchased from Bio X cell (Lebanon, NH, USA). Other reagents were of reagent grade and used as received.

### Preparation of radiolabeled compounds

Ga-DOTA-K([^211^At]APBA)-c(RGDfK) ([^211^At]**1**) was synthesized according to our previous report [24].

### Cellular uptake experiments

Cellular uptake experiments were performed according to a previous report with slight modifications [28]. Colon-26 cells were cultured in RPMI 1640 medium containing 10% fetal bovine serum (FBS, Sigma-Aldrich Co. LLC, St Lowis, MO, USA) on 6-well culture plates (containing 3 × 10^5^ cells/well) for 24 h using a humidified atmosphere (5% CO_2_) incubator at 37 °C. After the removal of the medium, a mixed solution of [^211^At]**1** and [^125^I]**2** (3.7 kBq/each tracer/well) in medium without FBS was added. The radiolabeling solutions were diluted in saline containing 0.05% (w/v) bovine serum albumin (BSA, Nacalai Tesque, Kyoto, Japan) to prevent adsorption to tubes, and further diluted with medium to 0.00125% as a final BSA concentration, which does not affect the cellular uptake of the radioligands [29]. After incubation for 1, 3, and 6 h, the medium from each well was removed, and the cells were washed twice with ice-cold PBS (−). The surface of the cells was washed twice with ice-cold 0.2 M glycine buffer (pH 3.0). The cells were lysed using 1 M NaOH aqueous solution. The radioactivity of ^125^I and ^211^At in 1 M NaOH was determined using an auto well gamma counter (ARC-7010, ALOKA CO., Ltd., Tokyo, Japan) according to a previous report [22] and defined as internalized radioligand fraction. The protein amount of cells was quantified using a BCA Protein Assay Kit (Nacalai Tesque) according to the manufacturer’s protocol. All data were expressed as percent dose per milligram protein (%dose/mg protein).

In blocking experiments, c(RGDfK) (final concentration 10 µM), which was synthesized according to a previous report [30], was added to each well with tracers. After incubation for 1, 3, and 6 h, radioactivity and protein concentration were determined using the abovementioned method.

### Animals

Experiments with animals were conducted in strict accordance with the Guidelines for the Care and Use of Laboratory Animals of Kanazawa University. The experimental protocols were approved by the Committee on Animal Experimentation of Kanazawa University. The animals were housed with free access to food and water at 23 °C with a 12-hour alternating light/dark schedule.

To prepare tumor-bearing mice, 1 × 10^6^ of cells (Colon-26) were subcutaneously inoculated into the right shoulder of 5-week-old female BALB/c mice (15–17 g, Japan SLC, Inc., Hamamatsu, Japan) or 5-week-old female BALB/c nu/nu mice (13–15 g, Japan SLC, Inc.). At 7 days postinoculation of Colon-26 cells, they were used for each experiment.

### Biodistribution experiments

[^211^At]**1** (37 kBq) and [^125^I]**2** (37 kBq) were intravenously coadministered into Colon-26 tumor-bearing BALB/c mice. Mice were sacrificed at 1, 4, and 24 h postinjection. Tissues of interest were removed and weighed, and the radioactivity in each tissue was determined. To determine the amount and routes of the radioactivity excreted from the body for 24 h after injection of [^211^At]**1** and [^125^I]**2**, mice were housed in metabolic cages (METABOLICA, SUGIYAMA-GEN Co., Ltd., Tokyo, Japan). Data are shown as the percentage of injected dose per gram of tissues (%ID/g) or %ID.

### Therapeutic experiments

Colon-26 tumor-bearing BALB/c mice were randomly divided into nine groups, the [^211^At]**1** (675 kBq)-treated group (*n* = 5), the [^211^At]**1** (675 kBq) + αPD-1-treated group (*n* = 12), the [^211^At]**1** (675 kBq) + αCTLA-4-treated group (*n* = 5), the [^211^At]**1** (270 kBq)-treated group (*n* = 4), the [^211^At]**1** (270 kBq) + αPD-1-treated group (*n* = 4), the [^211^At]**1** (270 kBq) + αCTLA-4-treated group (*n* = 5), the vehicle-treated group (*n* = 5), the αPD-1-treated group (*n* = 4), and the αCTLA-4-treated group (*n* = 5). The [^211^At]**1** (675 kBq) + αPD-1-treated group had a larger sample size than the other groups to confirm the predicted diversity of effects due to the combination with αPD-1. The maximum dose of [^211^At]**1** was set to 675 kBq because it is the maximum tolerated dose of [^211^At]**1** in U-87 MG tumor-bearing BALB/c nu/nu mice [31]. After 4 h of [^211^At]**1** intravenous administration, αPD-1 (10 mg/kg) or αCTLA-4 (10 mg/kg) was intravenously administered to the tumor-bearing mice. The doses of ICBs (αPD-1 and αCTLA-4) and the interval between [^211^At]**1** and ICB were set according to previous studies [17, 32].

Moreover, to confirm the difference of therapeutic effects derived from the antitumoral immune responses of [^211^At]**1**, [^211^At]**1** (675 kBq, *n* = 4), [^211^At]**1** (675 kBq) + αCTLA-4 (10 mg/kg) (*n* = 4), or vehicle (*n* = 3) was also administered to Colon-26 tumor-bearing BALB/c nu/nu mice. In the [^211^At]**1** (675 kBq) + αCTLA-4-treated group, αCTLA-4 was intravenously administered at 4 h postinjection of [^211^At]**1** as described above. Tumor volume and body weight of mice were monitored from 4 to 6 times weekly. Tumor size was measured with a slide caliper, and tumor volume was calculated using a formula: volume = 1/2 × length × width^2^ [33]. The tumor volume and body weight compared to the values on the day of the treatment (relative tumor volume). The mice were euthanized humanely when body weight was less than 80% at baseline (day 0), when the tumor weight reached more than 10% of body weight, or when the tumors became ulcerated as the endpoint.

### Hematoxylin and eosin (HE) staining and immunofluorescence staining

At 7 days postinjection of [^211^At]**1** (675 kBq) or vehicle, mice were sacrificed, and tumor tissues were collected. The tissues were fixed in a 4% paraformaldehyde in PBS (−) for 24 h at 4 °C, then cryoprotected in sucrose and embedded in O.C.T. compound (Sakura Finetek, Torrance, CA). Frozen tissues were sectioned at 5 μm thickness for HE staining and 15 μm thickness for immunofluorescence staining using a cryostat (Leica Biosystem, Nussloch, Germany). For HE staining, sections were stained with hematoxylin and eosin (Sakura Finetek) according to the manufacturer’s protocol and imaged using a BZ-X800 microscope (Keyence, Osaka, Japan). Immunofluorescence staining was performed according to a previous study [34]. Namely, sections were first treated with PBS (−) containing 1.5% BSA for 1 h at room temperature for blocking. After washing with PBS (−), the sections were stained with purified antimouse CD8a antibodies (clone 53−6.7, BioLegend, San Diego, CA, USA) as primary antibodies overnight at 4 °C, followed by staining with Anti-Rat IgG2a eFluor570 antibodies (clone r2a-21B2) (Thermo Fisher Scientific Inc., Waltham, MA, USA) as secondary antibodies for 2 h at room temperature. Nuclei were counter-stained with Hoechst 33342 (Nacalai Tesque). Fluorescent images were acquired using a confocal laser scanning microscopy (LSM710, Carl Zeiss, Oberkochen, Germany). Excitation wavelengths of 405 nm for Hoechst 33342 and 543 nm for eFluor 570 were used, with emission detected using filter ranges of 410–587 nm for Hoechst 33342 and 568–712 nm for eFluor 570, respectively.

### Flow cytometry

BALB/c mice bearing Colon-26 tumors were administered [^211^At]**1** (675 kBq) + αCTLA-4 (10 mg/kg), [^211^At]**1** (675 kBq), αCTLA-4 alone (10 mg/kg), or vehicle (*n* = 3 per group). In the combination group, αCTLA-4 was intravenously administered at 4 h postinjection of [^211^At]**1**. At 7 days treatment, mice were sacrificed, and tumor tissues were collected and minced in lysis buffer (150 mM NH_4_Cl, 10 mM NaHCO_3_, 0.1 mM EDTA). After filtration using the 40 μm filter, cells were suspended in PBS (−) for staining. Cells were stained with fluorophore-conjugated anti-mouse antibodies against CD4 (clone GK1.5, PE conjugated; BD Biosciences, San Jose, CA, USA), CD8a (clone 53−6.7, PE/Cyanine7 conjugated; BioLegend), and CD45R/B220 (clone RA3-6B2, FITC conjugated; BioLegend). After 30 min incubation at 4 °C, cells were washed with PBS (−), and incubated with 7-amino-actinomycin D (7-AAD) viability staining solution (BioLegend) for 10 min at room temperature. Stained cells were analyzed using a flow cytometer (RF-500, Sysmex Corporation, Kobe, Japan), and data were processed with FlowJo Software (v10.10.0, BD Biosciences). Geometric mean fluorescence intensity (gMFI) was used to quantify the expression level of cell surface markers, as it better represents fluorescence data with a log-normal distribution.

To investigate whether the enhanced therapeutic efficacy observed in some mice within the [^211^At]**1** (675 kBq) + αPD-1 (10 mg/kg) group was attributable to immune responses, a flow cytometric analysis of tumor cells was performed. In this experiment, αPD-1 was intravenously administered at 4 h postinjection of [^211^At]**1** (*n* = 11). Mice were sacrificed at day 16, when the differences in therapeutic efficacy were observed, and tumor tissues were analyzed as described above.

### Statistical evaluation

Double tracer experiments in the cellular uptake and biodistribution experiments were compared using paired Students’ *t* test. Blocking studies were compared using unpaired Students’ *t* test. Therapeutic experiments and flow cytometric analysis were analyzed by one-way analysis of variance (ANOVA) followed by Tukey-Kramer post hoc test. Kaplan–Meier survival curves were compared by Log-rank test, and pairwise comparisons were subsequently performed with Bonferroni correction for multiple testing. Given the large number of treatment groups, pairwise comparisons were selectively performed based on predefined hypotheses to focus on the following key questions: (1) dose-dependent effects of [^211^At]**1**, (2) additive effects of αPD-1, (3) additive effects of αCTLA-4, and (4) differences between immune checkpoint inhibitors. The level of statistical significance was set to a *p* < 0.05. All statistical analyses were conducted using GraphPad Prism version 10.5 (GraphPad Software, San Diego, CA, USA).

## Results

### Preparation of [^211^At]**1**

The radiochemical yield of [^211^At]**1** was 69%. After HPLC purification, the radiochemical purity was > 97%. HPLC purification completely separated the radiolabeled compounds from the precursor.

### Cellular uptake experiments

**Fig. 2:**
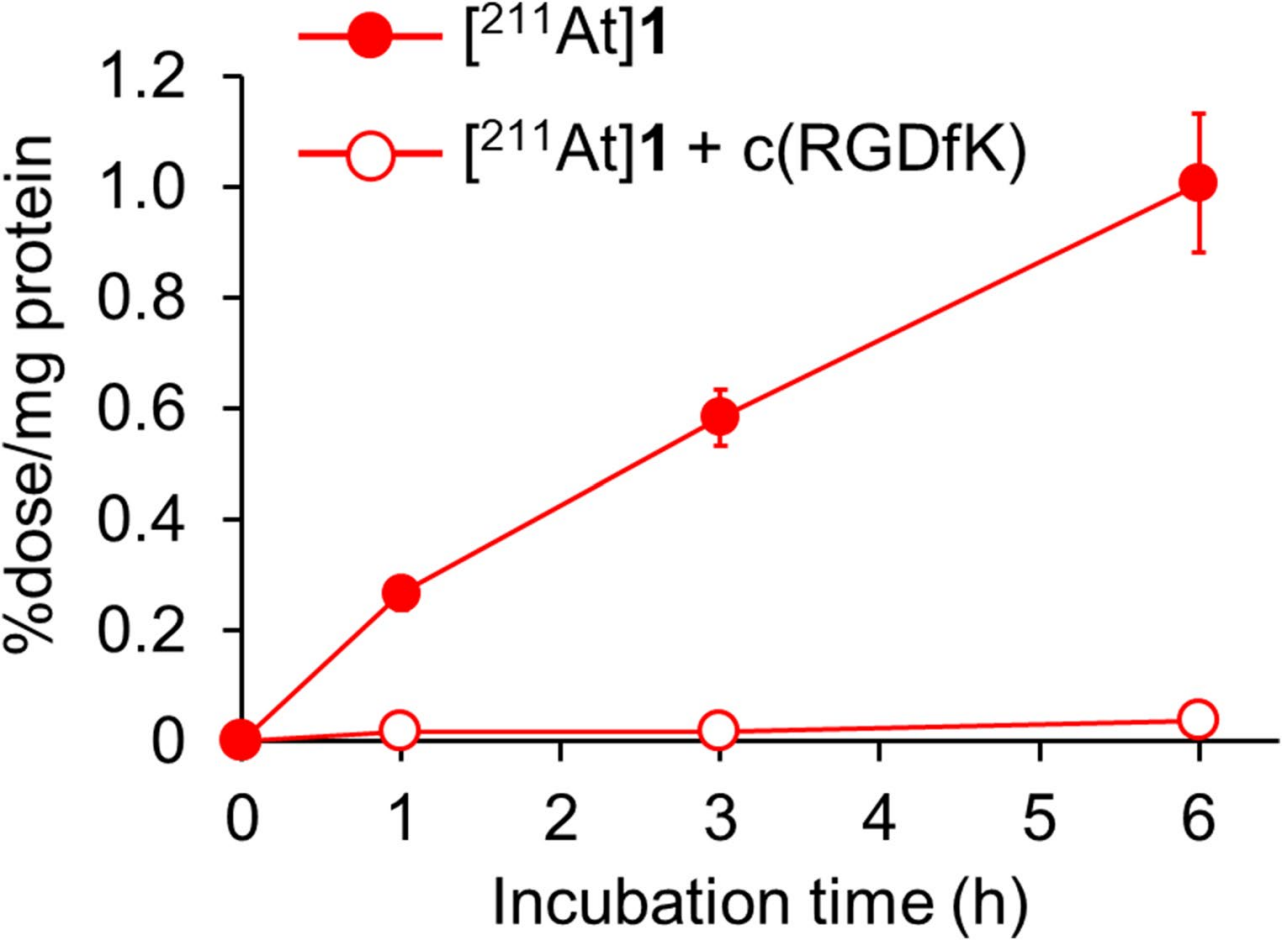
Cellular uptake experiments. Time-dependent accumulation of [^211^At]**1** in Colon-26 cells. Data were presented as mean ± SD for three samples

[^211^At]**1** accumulation in Colon-26 cells increased in a time-dependent manner (Fig. 2). The uptake of [^211^At]**1** was significantly reduced by adding excess amounts of the c(RGDfK) peptide, indicating that the uptake was caused by specific binding to α_v_β_3_ integrin (Fig. 2).

### Biodistribution experiments

**Fig. 3:**
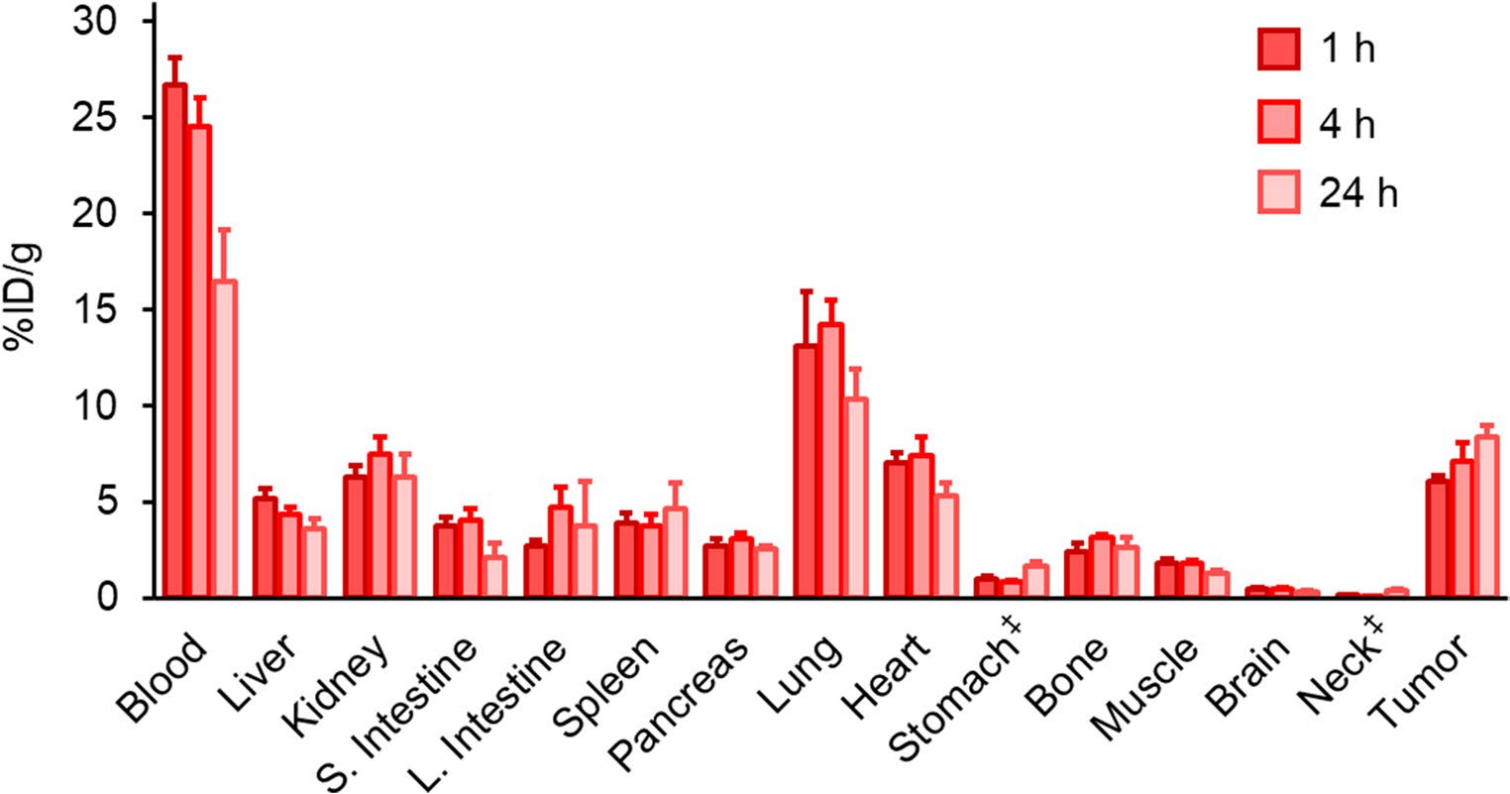
Biodistribution experiments. Biodistribution of radioactivity at 1, 4, and 24 h after concomitant intravenous injection of [^211^At]**1** in Colon-26 tumor-bearing BALB/c mice. ^‡^Expressed as % injected dose

Biodistribution experiments of [^211^At]**1** were conducted in Colon-26 tumor-bearing BALB/c mice. The results are shown in Fig. 3 and Table S1. [^211^At]**1** showed high blood retention and high tumor accumulation, was excreted mainly from the urine.

### Therapeutic experiments

**Fig. 4:**
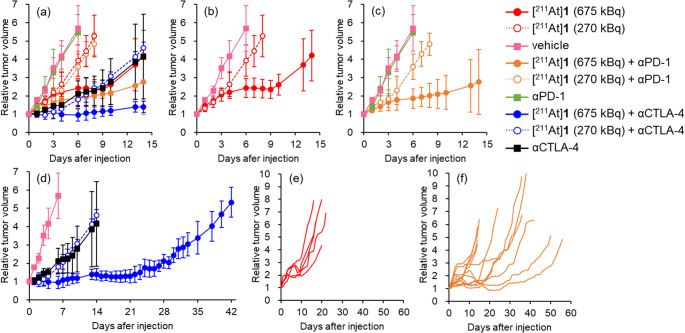
Therapeutic experiment in Colon-26 tumor-bearing BALB/c mice. Relative tumor volume in (**a**) all group, (**b**) [^211^At]**1** or vehicle-treated group, (**c**) [^211^At]**1** with or without αPD-1-treated group and vehicle-treated group, (**d**) [^211^At]**1** with or without αCTLA-4-treated group and vehicle-treated group (mean ± SD), (**e**) individual relative tumor volume in [^211^At]**1** (675 kBq)-treated group, (**f**) individual relative tumor volume in [^211^At]**1** (675 kBq) + αPD-1-treated group

**Fig. 5:**
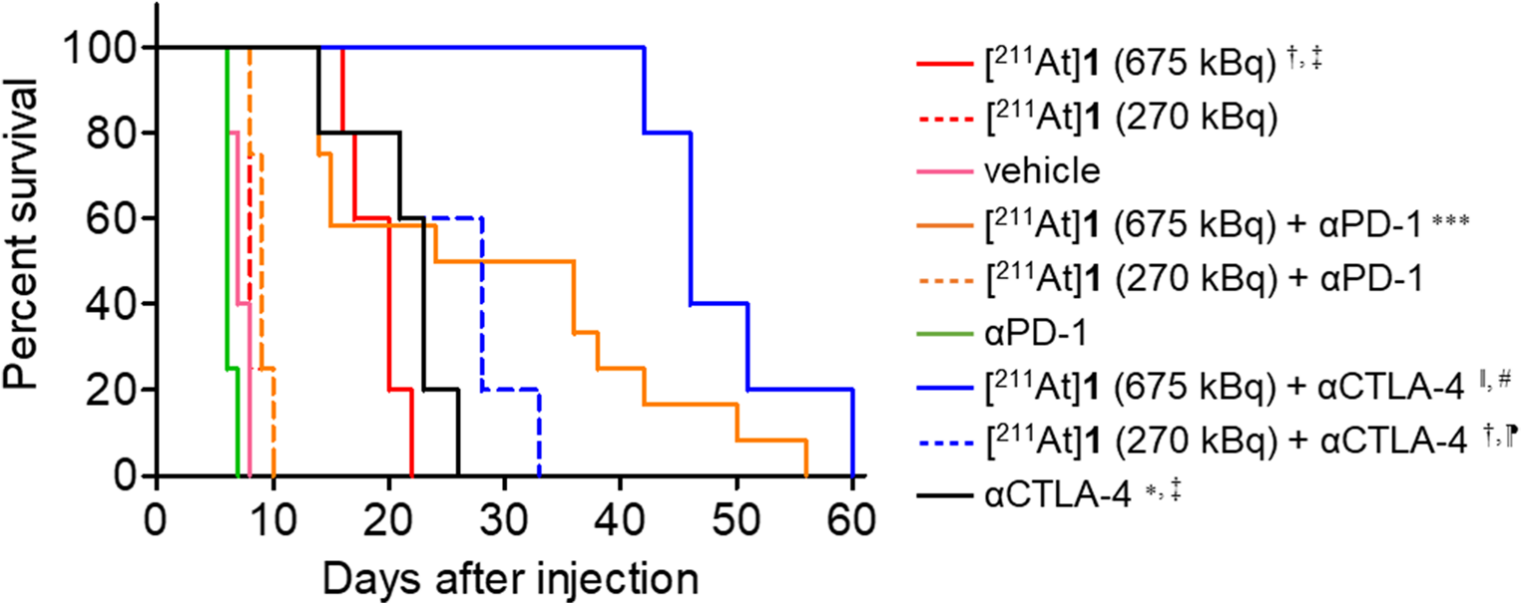
Kaplan-Meier survival curves. Statistical significance was determined by Log-rank test, and pairwise comparisons were subsequently performed with Bonferroni correction for multiple testing. ^†^*p* < 0.05 vs. [^211^At]**1** (270 kBq), ^‡^*p* < 0.05 vs. vehicle, ^*^p < 0.05 vs. αPD-1, ^***^p < 0.001 vs. αPD-1, ^‖^*p* < 0.05 vs. [^211^At]**1** (675 kBq), ^#^*p* < 0.05 vs. αCTLA-4, ^⁋^*p* < 0.05 vs. [^211^At]**1** (270 kBq) + αPD-1

The relative tumor volume of Colon-26 tumor-bearing BALB/c mice after treatment with two doses of [^211^At]**1** (270 or 675 kBq) or vehicle with or without ICB (αPD-1 or αCTLA-4, 10 mg/kg, respectively) are shown in Figs. 4a‒4d and Table S2. The [^211^At]**1** (675 kBq)-treated group showed significant inhibition of tumor growth compared with the [^211^At]**1** (270 kBq)-treated group and vehicle-treated group. The αPD-1-treated group or the [^211^At]**1** (270 kBq) + αPD-1-treated group did not significantly inhibit tumor growth compared with the vehicle-treated group or the [^211^At]**1** (270 kBq)-treated group, respectively. Although a trend toward tumor growth inhibition was observed in the [^211^At]**1** (675 kBq) + αPD-1-treated group compared to the [^211^At]**1** (675 kBq)-treated group, the difference was not statistically significant, likely due to considerable individual variability within the [^211^At]**1** (675 kBq) + αPD-1-treated group (Fig. 4e and f). Namely, 6 out of 12 mice in [^211^At]**1** (675 kBq) + αPD-1-treated group showed drastic tumor growth inhibition and prolonged survival (Figs. 4f and 5). However, the [^211^At]**1** (675 kBq) + αPD-1-treated group showed weight loss. Of the 12 mice in this group, 11 recovered, and 1 did not recover and was euthanized (Figures S2e and S2f). The αCTLA-4-treated group significantly inhibited tumor growth compared with both the vehicle-treated group and the [^211^At]**1** (270 kBq)-treated group, and its therapeutic efficacy was comparable to that of [^211^At]**1** (675 kBq). Although [^211^At]**1** (270 kBq) combined with αCTLA-4 did not enhance the therapeutic efficacy compared to αCTLA-4 alone, the [^211^At]**1** (675 kBq) + αCTLA-4-treated group exhibited the greatest therapeutic efficacy among all groups and resulted in the longest survival (Fig. 4a and d, and Fig. 5). However, this group also experienced weight loss, similar to the [^211^At]**1** (675 kBq) + αPD-1-treated group (Figure S2 and Table S3).

**Fig. 6:**
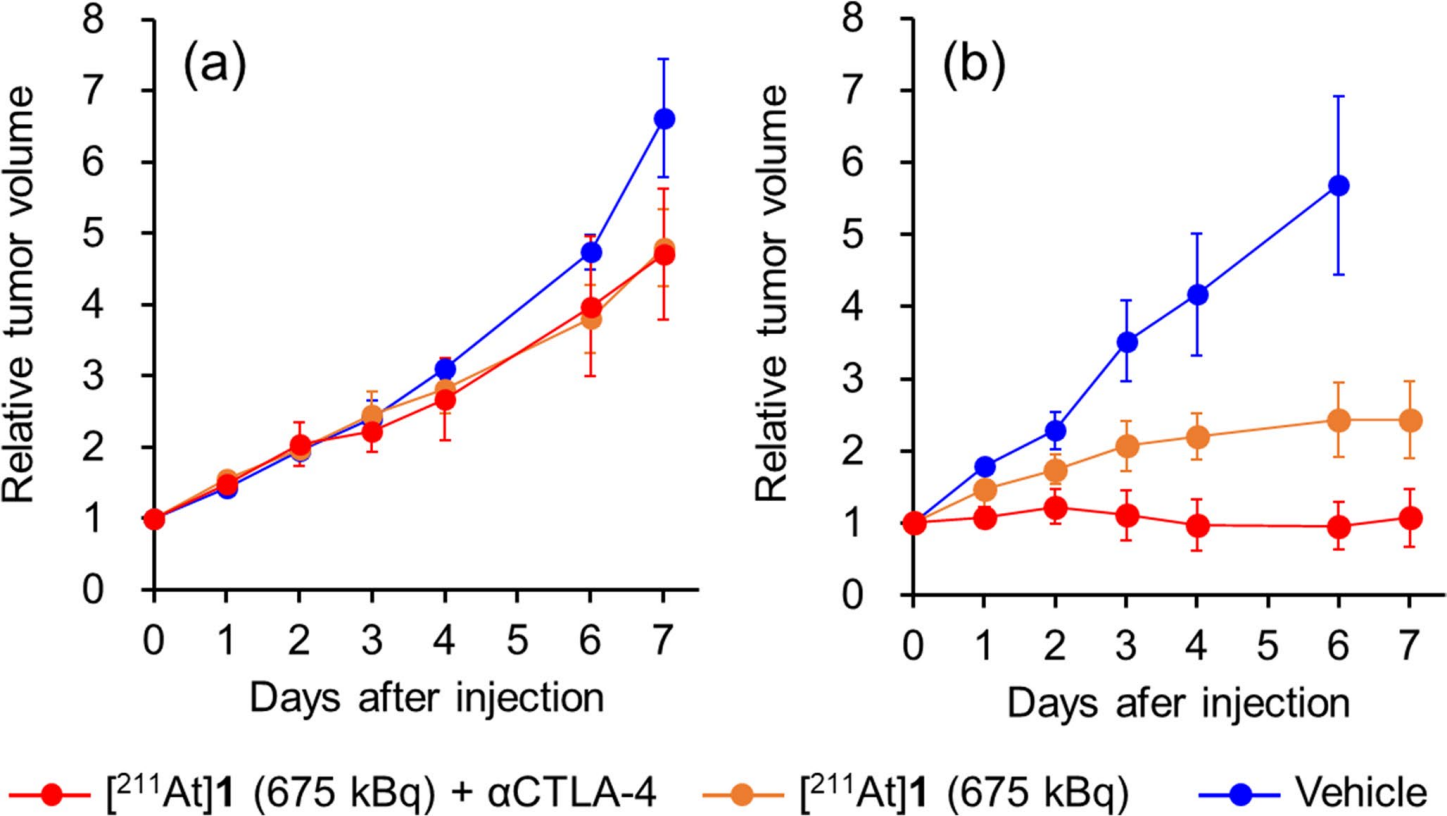
Therapeutic experiment in Colon-26 tumor-bearing mice. Tumor volume of (**a**) Colon-26 tumor-bearing BALB/c nu/nu mice and (**b**) Colon-26 tumor-bearing BALB/c mice after administration of [^211^At]**1** (675 kBq) + αCTLA-4, [^211^At]**1** (675 kBq), or vehicle. Data of (**b**) are reproduced from Fig. 4a. Data are expressed as relative values to initial tumor volume (mean ± SD)

The relative tumor volumes of Colon-26 tumor-bearing BALB/c nu/nu mice and BALB/c mice after treatment with [^211^At]**1** (675 kBq) + αCTLA-4, [^211^At]**1** (675 kBq), or vehicle are shown in Fig. 6, with detailed data provided in Tables S2 and S4. The tumor growth inhibition rate of [^211^At]**1** (675 kBq) on day 6 was lower in BALB/c nu/nu mice (20%) than in BALB/c mice (57%). In BALB/c mice, the combination therapy with [^211^At]**1** (675 kBq) and αCTLA-4 enhanced therapeutic efficacy, whereas no improvement was observed in BALB/c nu/nu mice.

### HE staining and infiltration of antitumor immune activity

**Fig. 7:**
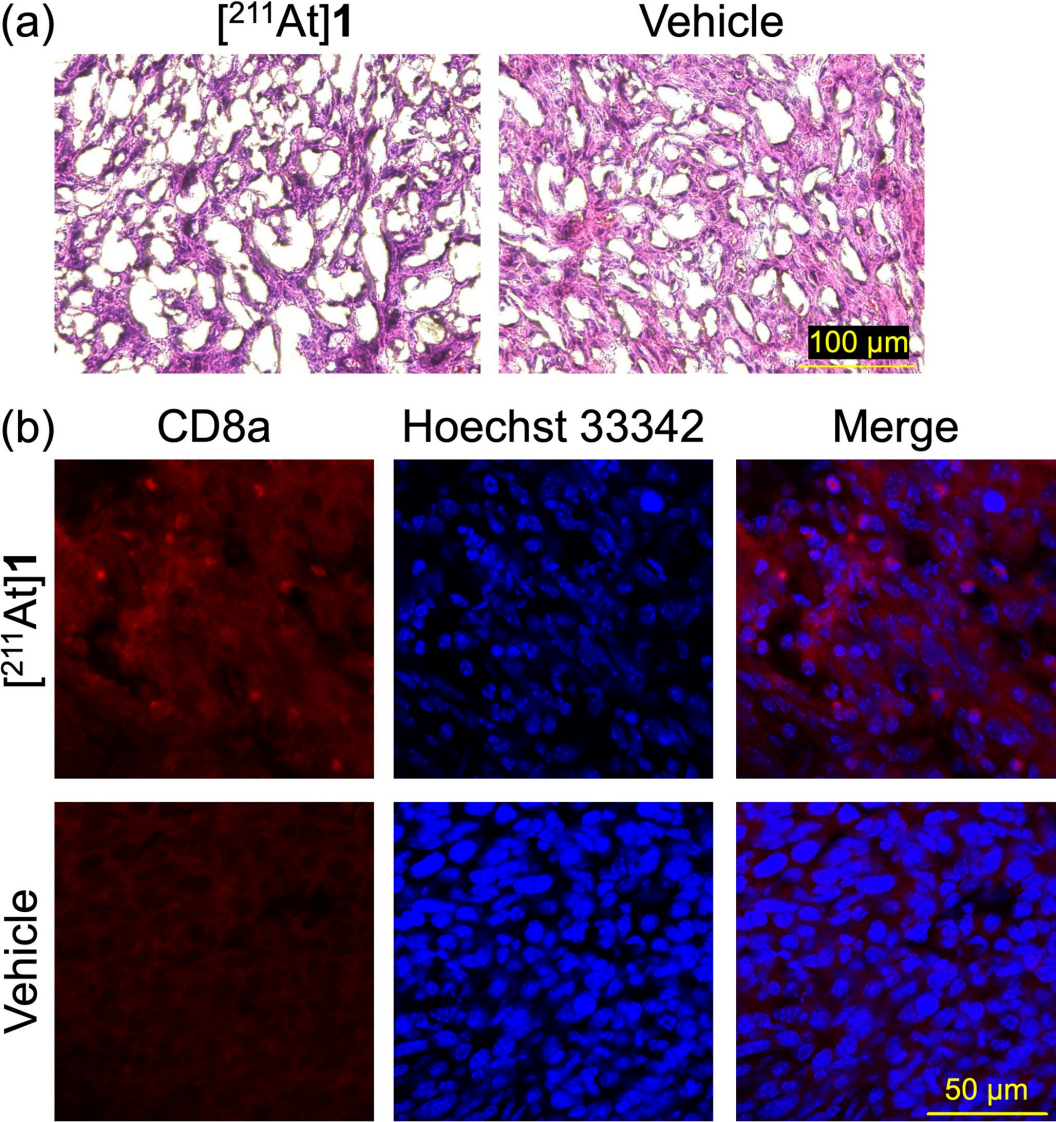
Histological and immunofluorescence analyses of Colon-26 tumors following [^211^At]**1** treatment. (**a**) HE staining. (**b**) immunofluorescence analysis. The fluorescence of CD8a is shown in red, and the nucleus stained with Hoechst 33342 is shown in blue

HE staining was conducted to evaluate tumor tissue damage. Following [^211^At]**1** administration, nuclear condensation and reduced tumor cell density were observed in HE-stained sections, indicating that α-particles emitted from [^211^At]**1** induced tumor tissue damages (Fig. 7a).

**Fig. 8:**
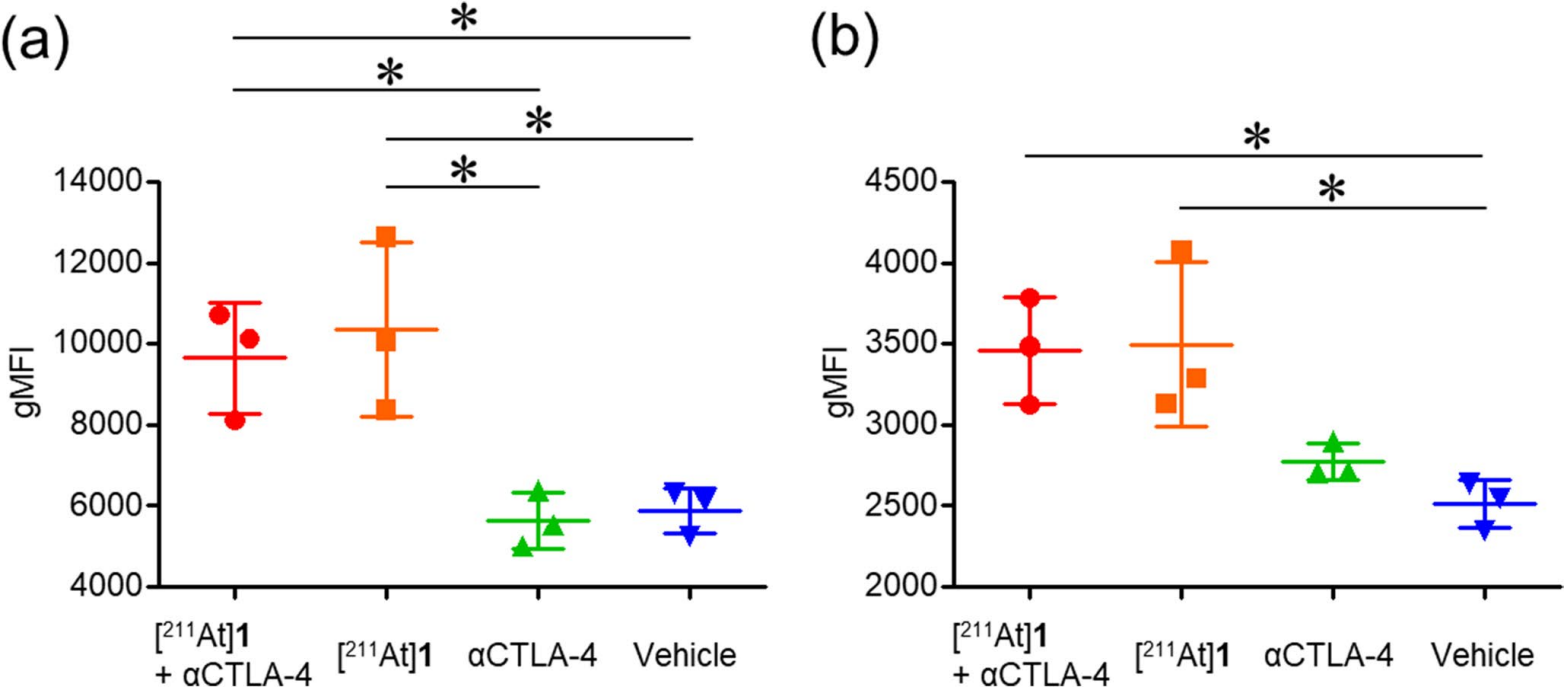
gMFI of (**a**) CD4^+^ T cells (CD4⁺CD45⁺ live cells) and (**b**) CD8^+^ T cells (CD8a⁺CD45⁺ live cells) in Colon-26 tumors assessed by flow cytometry on 7 days after treatment. Data are means ± SD (*n* = 3). Statistical significance was determined by ANOVA followed by Tukey-Kramer post hoc test (*p* < 0.05)

Figure 7b shows immunofluorescence staining of CD8a⁺ T cells (red), nuclei stained with Hoechst33342 (blue), and merged images in tumor sections from [²¹¹At]**1**- and vehicle-treated groups. T cell infiltration was observed by immunofluorescence staining of cancer tissue sections. In addition, flow cytometric analysis was performed to evaluate T cell infiltration quantitatively. In this analysis, gMFI values were measured for gated CD4⁺CD45⁺ and CD8a⁺CD45⁺ live cells as an indicator of T cell activation. At 7 days after [^211^At]**1** treatment, the activities of CD4^+^CD45^+^ helper T cells or regulatory T cells (CD4^+^ T cells) and CD8a^+^CD45^+^ cytotoxic T cells (CD8^+^ T cells) were significantly higher than those in the vehicle-treated group. However, the level of these immune cells of the [^211^At]**1** + αCTLA-4-treated group and the αCTLA-4-treated group did not significantly differ from those in the [^211^At]**1**-treated group and the vehicle-treated group, respectively (Fig. 8). These images indicate that increased infiltration of CD8a^+^ T cells was observed in the [^211^At]**1**-treated tumors.

As described above, a subset of mice treated with [^211^At]**1** (675 kBq) in combination with αPD-1 exhibited greater tumor growth suppression and prolonged survival compared to those treated with [^211^At]**1** alone. To elucidate the potential mechanisms underlying these interindividual differences in therapeutic response, flow cytometric analysis of tumor-infiltrating cells was performed. However, no correlation was observed between therapeutic efficacy and the activity of CD4^+^ and CD8^+^ T cells (Figure S3).

## Discussion

In this study, cellular uptake of [^211^At]**1** in Colon-26 cells was evaluated to assess α_v_β_3_ integrin-mediated uptake. The accumulation of radioactivity was increased in a time-dependent manner similar to that observed in U-87 MG cells [24], though slightly lower. Meanwhile, the uptake was significantly reduced by excess c(RGDfK) peptide, indicating specificity for α_v_β_3_ integrin. In biodistribution experiments in Colon-26 tumor-bearing BALB/c mice, [^211^At]**1** showed a similar biodistribution pattern to that in U-87 MG tumor-bearing BALB/c nu/nu mice [24]. A previous study reported that radiolabeled RGD peptides accumulated not only in α_v_β_3_ integrin-positive tumor cells but also in tumor neo-vasculature expressing α_v_β_3_ integrins [35]. This unique property of targeting tumor neo-vasculature is not shared by other tumor-targeting carriers [36]. Therefore, the ability of RGD peptides to accumulate in the tumor neo-vasculature could be beneficial for therapy with [^211^At]**1**, as its accumulation in the tumor neo-vasculature could potentially damage abnormal blood vessels.

In this study, [^125^I]**2** was evaluated to compare with [^211^At]**1**. [^125^I]**2** showed equivalent uptake in Colon-26 cells to [^211^At]**1**, and the accumulation of radioactivity in the cells increased in a time-dependent manner (Figure S1). Furthermore, it showed equivalent biodistribution in Colon-26 tumor-bearing BALB/c mice (Table S1). Equivalent biodistribution of diagnostic and therapeutic radiopharmaceuticals is important for radiotheranostics [37, 38]. Given that [^211^At]**1**, [^67/68^Ga]**2**, and [^123/124/125^I]**2** are expected to exhibit equivalent pharmacokinetics, the combination of [²¹¹At]**1** for TAT with corresponding imaging surrogates, such as [⁶⁸Ga]**2** or [^124^I]**2** for positron emission tomography (PET) and [^67^Ga]**2** or [¹²³I]**2** for single photon emission computed tomography (SPECT), represents a promising strategy for advancing radiotheranostics.

In therapeutic experiment in Colon-26 tumor-bearing BALB/c mice, the results of ICB monotherapy were consistent with the previous study that αPD-1 monotherapy had little therapeutic effects, however αCTLA-4 monotherapy had high therapeutic effects [39]. Meanwhile, the observed difference in therapeutic response between the αCTLA-4- and αPD-1-treated groups could be attributed to the characteristics of the Colon-26 tumor-bearing model, including its immune microenvironment and checkpoint expression profile. The therapeutic efficacy of [^211^At]**1** (270 kBq) was slight, suggesting that it may not cause sufficient damage to the tumor to trigger antitumor immune responses. Thus, combination therapy of [^211^At]**1** (270 kBq) and ICB (αPD-1 or αCTLA-4) did not enhance the therapeutic effect. However, the [^211^At]**1** (675 kBq)-treated group and [^211^At]**1** (675 kBq) + ICB (αPD-1 or αCTLA-4)-treated group showed significant tumor growth inhibition compared with the vehicle-treated control group. Although no statistically significant difference was observed between the [^211^At]**1** (675 kBq)-treated group and the [^211^At]**1** (675 kBq) and αPD-1 group because of large individual differences, higher tumor growth inhibition and prolonged survival were observed in some mice (6/12) in the combination therapy ([^211^At]**1** (675 kBq) and αPD-1) group (Fig. 4f). Previous studies have shown the effectiveness of the combination of TRT agents and ICB with individual differences [13, 14, 17]. Additionally, combination therapy of other therapies, such as radiotherapy [40, 41], photodynamic therapy [42, 43], and photoimmunotherapy [44, 45], with ICB has been reported to be effective with individual differences. In contrast, the [^211^At]**1** (675 kBq) + αCTLA-4-treated group exhibited the greatest therapeutic efficacy and the longest survival among all groups, and showed less inter-individual variability, unlike the [^211^At]**1** (675 kBq) + αPD-1-treated group. These findings suggest that the observed synergistic antitumor effect is attributable to the combined therapeutic activity of [^211^At]**1** (675 kBq) and αCTLA-4.

Meanwhile, mice treated with [^211^At]**1** (675 kBq) in combination with either αPD-1 or αCTLA-4 experienced greater body weight loss compared to those receiving monotherapy. Severe weight loss was observed in 1 out of 12 mice treated with [^211^At]**1** (675 kBq) + αPD-1. The enhanced weight loss following combination therapy with TAT and ICB has been previously reported and is presumed to be associated with cytokine storm induced by immune activation [20]. In this study, a single protocol was used, where ICB was administered 4 h postinjection of [^211^At]**1**. In contrast, some previous studies have employed repeated administration of ICB after TRT and reported higher therapeutic effects than TRT alone, with little weight loss or recovery if weight loss occurred [13, 14]. Therefore, optimizing the dosing schedule can enhance the therapeutic efficacy and reduce adverse effects. This raises concerns about the safety of such combination therapy in clinical translation, and further investigations are warranted to elucidate the underlying mechanisms and improve tolerability.

To address this issue, we previously developed a method to optimize the pharmacokinetics of [^211^At]**1**, namely, accumulation of [^211^At]**1** in the tumor, followed by administration of an albumin-binding inhibitor to accelerate the [^211^At]**1** clearance [31]. This method could reduce adverse effects while maintaining the therapeutic efficacy of [^211^At]**1** and ICB combination therapy.

Figure 6 demonstrates that the therapeutic effects of [^211^At]**1** in BALB/c mice were greater than those in BALB/c nu/nu mice. Moreover, the combination with αCTLA-4 further enhanced the therapeutic effect in BALB/c mice, whereas no enhanced therapeutic effect was observed in BALB/c nu/nu mice. These findings indicate that tumor growth inhibition in BALB/c mice is due to not only the cytotoxic effects of [^211^At]**1** itself but also antitumor immune responses. The limited therapeutic benefit in BALB/c nu/nu mice, which lack mature T cells, highlights the essential role of adaptive immune responses in mediating the antitumor effects of this therapeutic strategy for combination with TAT and ICB. As a factor involved in tumor progression, Galectin-1 (Gal-1) plays a role in cancer immune escape and induces T-cell apoptosis. AP-74 M-545, which interacts with Gal-1 and CD45, can protect T cells from apoptosis, resulting in T-cell-mediated immune responses. In a previous study, AP-74 M-545 showed no effect in immunodeficient mice due to the lack of T cells but showed efficacy in immunocompetent mice [46].

To evaluate immune cell infiltration in Colon-26 tumors following [^211^At]**1** (675 kBq) administration, flow cytometric analysis of CD4^+^ and CD8^+^ T cells and immunofluorescence staining of CD8a^+^ cells, representative immune cell subsets [14, 20], were performed. The results demonstrated infiltration of CD4^+^ and CD8^+^ T cells at 7 days postinjection of [^211^At]**1** (675 kBq) (Figs. 7b and 8). In addition, HE staining revealed that [^211^At]**1** induced tumor tissue damage (Fig. 7a). These findings support the notion that the combination therapy with [^211^At]**1** (675 kBq) and αCTLA-4 enhances therapeutic efficacy compared to [^211^At]**1** (675 kBq) monotherapy, potentially through an augmented antitumor immune response induced by tumor damage. Although combination therapy with [^211^At]**1** and αCTLA-4 enhanced therapeutic efficacy, αCTLA-4 did not induce tumor infiltration of CD4^+^ or CD8^+^ T cells (Fig. 8). This apparent discrepancy may not be contradictory, as several studies have reported that ICBs primarily reinvigorate the function of pre-existing tumor-infiltrating T cells rather than promoting their recruitment [47–49]. Therefore, the enhanced efficacy observed with combination therapy may reflect the qualitative improvement in T cell function rather than an increase in their number.

One possible explanation for the observed immune-related changes is the induction of ICD by [^211^At]**1**. While radiotherapy in general is known to release DAMPs [3, 4, 7, 8], α-particle irradiation could cause DNA double-strand breaks and cellular stress, leading to the release of DAMPs [50–52]. These immunostimulatory signals that enhance antigen presentation and T cell activation [12]. Although ICD markers were not directly assessed in this study, the increased CD8⁺ T cell infiltration following [^211^At]**1** treatment supports the hypothesis that ICD may contribute to the antitumor immune response. Future studies should evaluate DAMP expression and dendritic cell activation to validate ICD in [^211^At]**1** induced immunity, potentially providing a rationale for combining TAT with immunoadjuvants or ICD enhancers.

## Conclusion

[^211^At]**1** induced an antitumor immune response. To the best of our knowledge, this is the first study to intravenously administer a TAT agent ([^211^At]**1**), which is a clinically applicable method for various cancers, and to demonstrate that the combination of [^211^At]**1** with ICB enhances the therapeutic efficacy. Further studies are needed to optimize the dosing schedule to improve therapeutic efficacy.

## Supplementary Information

Below is the link to the electronic supplementary material.

Supplementary Material 1 Preparation method of Ga-DOTA-K([^125^I]IPBA)-c(RGDfK) ([^125^I]**2**), cellular uptake data of [^125^I]**2**, detailed biodistribution data, detailed therapeutic experiment data, and flow cytometric analysis data of Colon-26 tumors treated with [^211^At]**1** (675 kBq) + αPD-1.

## Data Availability

Data are available from the corresponding author on reasonable request.
