# Supplementary material for: Combination therapy of an 211At-labeled RGD peptide and immune checkpoint blockade to enhance antitumor efficacy
Source: Eur J Nucl Med Mol Imaging. 2025 Aug 6;53(2):992–1003. doi: 10.1007/s00259-025-07498-3 (PMC12830475; doi:10.1007/s00259-025-07498-3)
Supplement: Supplementary file 1 — Supplementary Material 1 Preparation method of Ga-DOTA-K([125I]IPBA)-c(RGDfK) ([125I]2), cellular uptake data of [125I]2, detailed biodistribution data, detailed therapeutic experiment data, and flow cytometric analysis data of Colon-26 tumors treated with [211At]1 (675 kBq) + αPD-1. [file 259_2025_7498_MOESM1_ESM.docx]

**Supporting Information**

**Combination therapy of an ^211^At-labeled RGD peptide and immune checkpoint blockade to enhance antitumor efficacy**

Hiroaki Echigo^a^, Masayuki Munekane^a^, Takeshi Fuchigami^a^, Kohshin Washiyama^b^, Takashi Nakamura^a^, Atsushi Furukawa^a^, Zhuoqing Chen^c^, Kenji Mishiro^a^, Hiroshi Wakabayasi^c^, Kazuhiro Takahashi^b^, Seigo Kinuya^c^, Kazuma Ogawa*^, a^

^a^ *Graduate School of Medical Sciences, Kanazawa University, Kakuma-machi, Kanazawa, Ishikawa 920-1192, Japan;*

^b^ *Advanced Clinical Research Center, Fukushima Global Medical Science Center, Fukushima Medical University, 1 Hikarigaoka, Fukushima, 960-1295, Japan;*

^c^ *Department of Nuclear Medicine, Kanazawa University Hospital, Kanazawa University, Takara-machi 13-1, Kanazawa, Ishikawa 920-8641, Japan;*

***Corresponding Author**

Institute for Frontier Science Initiative; Kanazawa University; Kakuma-machi, Kanazawa 920-1192; Japan.

Telephone: +81-76-234-4460; Fax: +81-76-234-4460

E-mail: [kogawa@p.kanazawa-u.ac.jp](mailto:kogawa@p.kanazawa-u.ac.jp)

**Table of contents**

Preparation of Ga-DOTA-K([^125^I]PBA)-c(RGDfK) ([^125^I]**2**)S3

Figure S1 Cellular uptake experiments of [^125^I]**2**S3

Table S1 Biodistribution of [^211^At]**1** and [^125^I]**2** in Colon-26 tumor-bearing BALB/c miceS4

Figure S2 Relative body weight of Colon-26 tumor-bearing BALB/c mice in therapeutic experimentS6

Table S2 Detailed relative tumor volume of Colon-26 tumor-bearing BALB/c mice in therapeutic experiment S7

Table S3 Detailed relative body weight of Colon-26 tumor-bearing BALB/c mice in therapeutic experimentS8

Table S4 Detailed relative tumor volume of Colon-26 tumor-bearing nu/nu mice in therapeutic experimentS9

Table S5 Detailed relative body weight of Colon-26 tumor-bearing nu/nu mice in therapeutic experiment S9

Figure S3 Flow cytometric analysis of Colon-26 tumors treated with [^211^At]**1** + αPD-1S10

ReferenceS11

**Preparation of** [^125^I]**2**

[^125^I]Sodium iodide (629 GBq/mg) was purchased from Revvity Japan (Yokohama, Japan). Ga-DOTA-K([^125^I]IPBA)-c(RGDfK) ([^125^I]**2**) were synthesized according to our previous report [1]. The radiochemical yield of [^125^I]**2** was 54%. After HPLC purification, the radiochemical purity was > 97%. HPLC purification completely separated the radiolabeled compounds from the precursor.

**
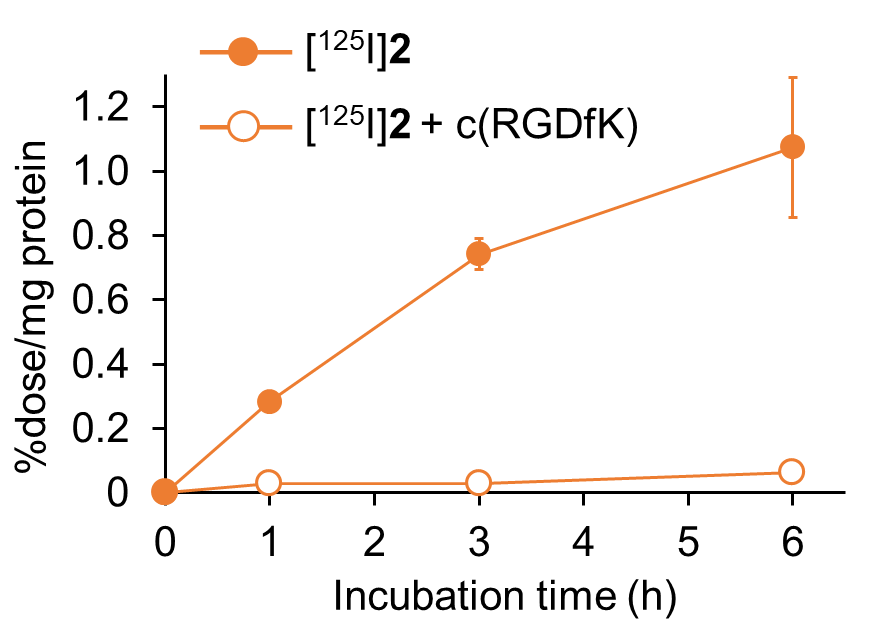
**

**Figure S1.** Cellular uptake experiments. Time-dependent accumulation of [^125^I]**2** in Colon-26 cells. Data were presented as mean ± SD for three samples.

**Table S1.** Biodistribution of radioactivity after intravenous injection of [^211^At]**1** and [^125^I]**2** in Colon-26 tumor-bearing BALB/c mice.

| Tissues | Times after injection | | |
| --- | --- | --- | --- |
|  | 1 h | 4 h | 24 h |
| [^211^At]**1** |  |  |  |
| Blood | 26.71 (1.45) | 24.54 (1.49) | 16.47 (2.71) |
| Liver | 5.21 (0.53) | 4.39 (0.36) | 3.63 (0.51) |
| Kidney | 6.34 (0.56) | 7.51 (0.87) | 6.27 (1.22) |
| S. intestine | 3.78 (0.46) | 4.08 (0.60) | 2.10 (0.75) |
| L. intestine | 2.70 (0.31) | 4.75 (1.02) | 3.77 (2.34) |
| Spleen | 3.88 (0.54) | 3.80 (0.59) | 4.70 (1.32) |
| Pancreas | 2.70 (0.37) | 3.10 (0.29) | 2.55 (0.18) |
| Lung | 13.13 (2.84) | 14.21 (1.31) | 10.38 (1.54) |
| Heart | 7.08 (0.48) | 7.46 (0.95) | 5.34 (0.68) |
| Stomach^‡^ | 1.01 (0.14) | 0.88 (0.04) | 1.70 (0.22) |
| Bone | 2.41 (0.49) | 3.16 (0.16) | 2.61 (0.52) |
| Muscle | 1.84 (0.24) | 1.84 (0.11) | 1.34 (0.10) |
| Brain | 0.48 (0.07) | 0.47 (0.08) | 0.33 (0.08) |
| Neck^‡^ | 0.16 (0.02) | 0.09 (0.05) | 0.39 (0.12) |
| Tumor | 6.09 (0.31) | 7.12 (0.95) | 8.40 (0.60) |
| Urine^‡^ |  |  | 12.46 (3.08) |
| Feces^‡^ |  |  | 1.50 (0.58) |
|  |  |  |  |
| [^125^I]**2** |  |  |  |
| Blood | 25.77 (1.50) ^**^ | 21.84 (1.32) ^**^ | 12.57 (2.36) ^**^ |
| Liver | 5.00 (0.48) ^*^ | 4.01 (0.28) ^**^ | 2.81 (0.40) ^**^ |
| Kidney | 6.39 (0.50) | 7.25 (0.78) | 5.90 (1.16) |
| S. intestine | 3.50 (0.49) ^**^ | 3.84 (0.59) ^**^ | 1.91 (1.27) |
| L. intestine | 2.63 (0.30) | 4.83 (1.01) | 3.13 (1.56) |
| Spleen | 3.27 (0.54) ^*^ | 3.27 (0.48) ^*^ | 3.34 (0.97) ^*^ |
| Pancreas | 2.55 (0.35) ^*^ | 2.97 (0.73) | 1.91 (0.04) ^*^ |
| Lung | 12.45 (2.90) ^**^ | 12.85 (1.21) ^**^ | 7.46 (1.28) ^**^ |
| Heart | 6.90 (0.58) | 6.57 (0.78) ^**^ | 3.91 (0.64) ^**^ |
| Stomach^‡^ | 0.72 (0.13) ^**^ | 0.71 (0.06) ^*^ | 0.51 (0.07) ^*^ |
| Bone | 2.26 (0.42) | 2.55 (0.19) | 1.77 (0.18) |
| Muscle | 1.84 (0.17) | 1.67 (0.12) | 1.03 (0.10) ^**^ |
| Brain | 0.46 (0.07) | 0.41 (0.06) ^**^ | 0.25 (0.06) ^*^ |
| Neck^‡^ | 0.15 (0.03) | 0.10 (0.02) | 0.09 (0.02) ^*^ |
| Tumor | 6.31 (0.50) | 6.94 (1.03) | 6.68 (0.40) ^*^ |
| Urine^‡^ |  |  | 25.39 (4.84) ^*^ |
| Feces^‡^ |  |  | 1.65 (0.69) |

Expressed as % injected dose per gram.

Each value represents the mean (SD) for three or four animals.

^‡^ Expressed as % injected dose.

Significance was determined by paired *t* test.

^*^*p* < 0.05, ^**^*p* < 0.01 vs [^211^At]**1**.

**
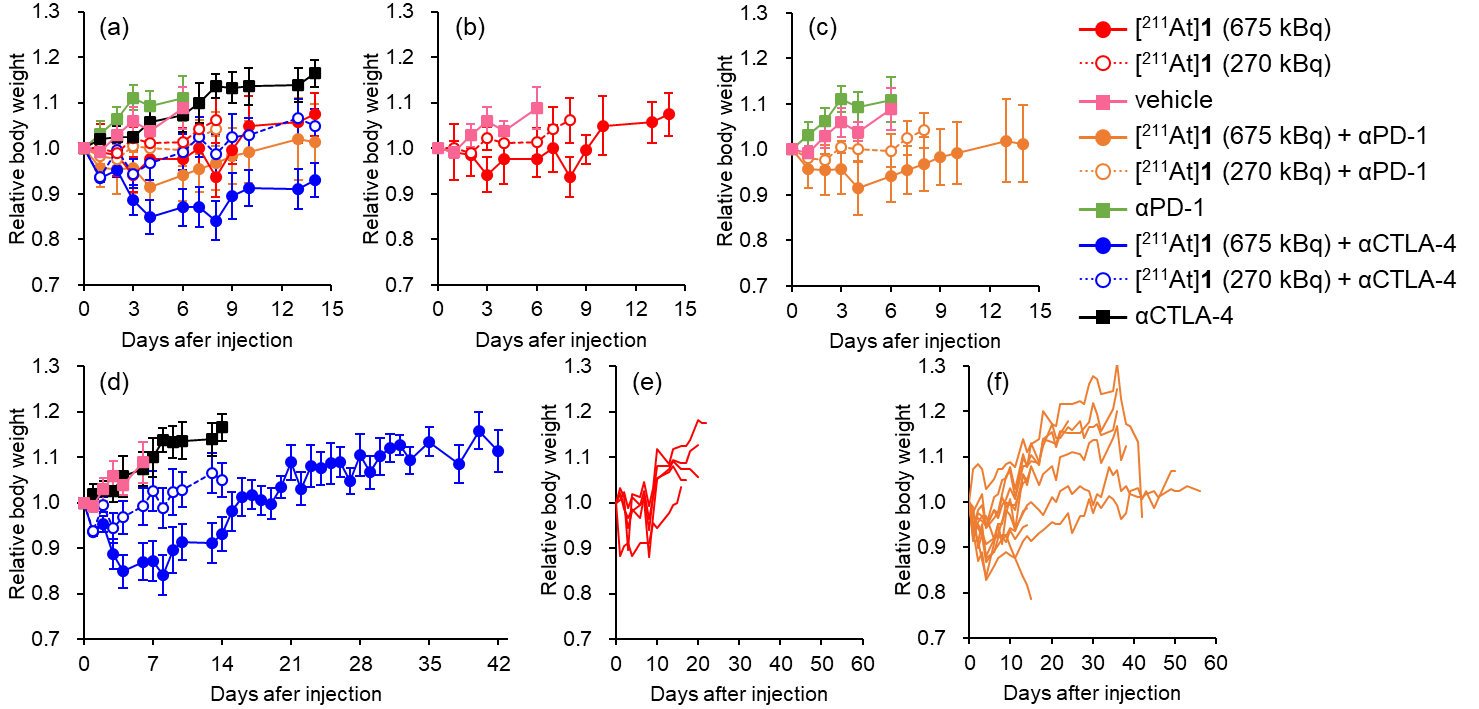
**

**Figure S2.** Therapeutic experiment in Colon-26 tumor-bearing BALB/c mice. Relative body weight in (a) all group, (b) [^211^At]**1** or vehicle-treated group, (c) [^211^At]**1** with or without αPD-1-treated group, (d) [^211^At]**1** with or without αCTLA-4-treated group (mean ± SD), (e) individual relative body weight in [^211^At]**1** (675 kBq) treated group, (f) individual relative body weight in [^211^At]**1** (675 kBq) + αPD-1 treated group.

**Table S2.** Relative tumor volume of Colon-26 tumor-bearing BALB/c mice after administration of [^211^At]**1** (270 or 675 kBq) with αPD-1 or αCTLA-4, [^211^At]**1** (270 or 675 kBq), αPD-1, αCTLA-4, or vehicle. Data are expressed as relative value to initial tumor volume (mean ± SD).

| Days after  injection | [^211^At]**1**  (675 kBq)  + αPD-1 | [^211^At]**1**  (675 kBq)  + αCTLA-4 | [^211^At]**1**  (675 kBq) | [^211^At]**1**  (270 kBq)  + αPD-1 | [^211^At]**1**  (270 kBq)  + αCTLA-4 | [^211^At]**1**  (270 kBq) | αPD-1 | αCTLA-4 | vehicle |
| --- | --- | --- | --- | --- | --- | --- | --- | --- | --- |
| 0 | 1.00 (0.00) | 1.00 (0.00) | 1.00 (0.00) | 1.00 (0.00) | 1.00 (0.00) | 1.00 (0.00) | 1.00 (0.00) | 1.00 (0.00) | 1.00 (0.00) |
| 1 | 1.23 (0.14) ^a^ | 1.08 (0.14) ^a, g^ | 1.47 (0.30) ^b, e^ | 1.41 (0.18) ^e^ | 0.97 (0.07) ^a, c^ | 1.32 (0.18) ^a^ | 1.44 (0.28) ^b^ | 1.05 (0.10) ^a^ | 1.79 (0.06) |
| 2 | 1.41 (0.24) ^a, c^ | 1.22 (0.24) ^a, c^ | 1.74 (0.21) ^e^ | 1.66 (0.32) ^c, e^ | 0.97 (0.15) ^a, c, d^ | 1.66 (0.29) ^c^ | 2.36 (0.70) ^b^ | 1.24 (0.14) ^a^ | 2.28 (0.26) |
| 3 | 1.65 (0.45) ^a, c^ | 1.11 (0.35) ^a, c^ | 2.07 (0.35) ^a, c^ | 1.85 (0.48) ^a, c^ | 1.14 (0.27) ^a, c^ | 2.20 (0.20) ^a^ | 3.28 (1.26) ^b^ | 1.44 (0.27) ^a^ | 3.52 (0.56) |
| 4 | 1.77 (0.53) ^a, c^ | 0.97 (0.36) ^a, c, d, f, g^ | 2.20 (0.32) ^a, c^ | 2.31 (0.78) ^a, c^ | 1.29 (0.48) ^a, c, d^ | 2.63 (0.65) ^a, b^ | 4.10 (0.64) ^b^ | 1.51 (0.22) ^a^ | 4.17 (0.84) |
| 6 | 1.84 (0.50) ^a, c, d, f^ | 0.96 (0.33) ^a, c, d, f^ | 2.43 (0.52) ^a, c^ | 3.59 (0.70) ^a, c, e^ | 1.81 (1.15) ^a, c, d^ | 3.93 (0.86) ^a, b^ | 5.45 (0.99) ^b^ | 2.12 (0.70) ^a^ | 5.68 (1.23) |

Data are expressed as relative value to initial tumor volume mean (SD).

Significance was determined by one-way analysis of variance followed by Tukey-Kramer post hoc test.

^a^*p* < 0.05 vs vehicle, ^b^*p* < 0.05 vs αCTLA-4, ^c^*p* < 0.05 vs αPD-1, ^d^*p* < 0.05 vs [^211^At]**1** (270 kBq), ^e^*p* < 0.05 vs [^211^At]**1** (270 kBq) + αCTLA-4, ^f^*p* < 0.05 vs [^211^At]**1** (270 kBq) + αPD-1, ^g^*p* < 0.05 vs [^211^At]**1** (675 kBq).

**Table S3.** Relative body weight of Colon-26 tumor-bearing BALB/c mice after administration of[^211^At]**1** (270 or 675 kBq) with αPD-1 or αCTLA-4, [^211^At]**1** (270 or 675 kBq), αPD-1, αCTLA-4, or vehicle. Data are expressed as relative value to initial tumor volume (mean ± SD).

| Days after  injection | [^211^At]**1**  (675 kBq)  + αPD-1 | [^211^At]**1**  (675 kBq)  + αCTLA-4 | [^211^At]**1**  (675 kBq) | [^211^At]**1**  (270 kBq)  + αPD-1 | [^211^At]**1**  (270 kBq)  + αCTLA-4 | [^211^At]**1**  (270 kBq) | αPD-1 | αCTLA-4 | vehicle |
| --- | --- | --- | --- | --- | --- | --- | --- | --- | --- |
| 0 | 1.00 (0.00) | 1.00 (0.00) | 1.00 (0.00) | 1.00 (0.00) | 1.00 (0.00) | 1.00 (0.00) | 1.00 (0.00) | 1.00 (0.00) | 1.00 (0.00) |
| 1 | 0.96 (0.04) ^b, c^ | 0.93 (0.01) ^b, c^ | 0.99 (0.06) | 0.98 (0.02) | 1.00 (0.02) ^b, c^ | 1.00 (0.02) | 1.03 (0.03) | 1.02 (0.02) | 0.99 (0.01) |
| 2 | 0.95 (0.06) ^a, b, c^ | 0.95 (0.02) ^a, c^ | 0.98 (0.05) | 0.98 (0.01) ^c^ | 0.99 (0.01) | 0.99 (0.01) | 1.06 (0.03) | 1.03 (0.02) | 1.03 (0.03) |
| 3 | 0.96 (0.05) ^a, b, c, h^ | 0.89 (0.03) ^a, b, c, d, f^ | 0.94 (0.04) ^a, b, c^ | 1.00 (0.01) ^c^ | 1.02 (0.03) ^a, b, c^ | 1.02 (0.03) ^c^ | 1.11 (0.03) ^b^ | 1.03 (0.02) | 1.06 (0.03) |
| 4 | 0.91 (0.06) ^a, b, c, d^ | 0.85 (0.04) ^a, b, c, d, e, f, g^ | 0.98 (0.05) ^c^ | 1.00 (0.03) | 1.01 (0.03) ^c^ | 1.01 (0.03) | 1.09 (0.03) | 1.06 (0.05) | 1.04 (0.02) |
| 6 | 0.94 (0.06) ^a, b, c^ | 0.87 (0.04) ^a, b, c, d, e, f, g^ | 0.98 (0.04) ^a, b, c^ | 1.00 (0.04) ^c^ | 1.01 (0.03) ^a, c^ | 1.01 (0.03) | 1.11 (0.05) | 1.07 (0.04) | 1.09 (0.05) |

Data are expressed as relative value to initial body weight mean (SD).

Significance was determined by one-way analysis of variance followed by Tukey-Kramer post hoc test.

^a^*p* < 0.05 vs vehicle, ^b^*p* < 0.05 vs αCTLA-4, ^c^*p* < 0.05 vs αPD-1, ^d^*p* < 0.05 vs [^211^At]**1** (270 kBq), ^e^*p* < 0.05 vs [^211^At]**1** (270 kBq) + αCTLA-4, ^f^*p* < 0.05 vs [^211^At]**1** (270 kBq) + αPD-1, ^g^*p* < 0.05 vs [^211^At]**1** (675 kBq), ^h^*p* < 0.05 vs [^211^At]**1** (675 kBq) + αCTLA-4.

**Table S4.** Relative tumor volume of Colon-26 tumor-bearing BALB/c nu/nu mice after administration of [^211^At]**1** (675 kBq) + αCTLA-4, [^211^At]**1** (675 kBq), or vehicle. Data are expressed as relative value to initial tumor volume (mean ± SD).

| Days after injection | [^211^At]**1 (**675 kBq)  +αCTLA-4 | [^211^At]**1** 675 kBq | Vehicle |
| --- | --- | --- | --- |
| 0 | 1.00 (0.00) | 1.00 (0.00) | 1.00 (0.00) |
| 1 | 1.48 (0.09) | 1.56 (0.07) | 1.43 (0.02) |
| 2 | 2.05 (0.31) | 1.98 (0.14) | 1.95 (0.09) |
| 3 | 2.23 (0.30) | 2.46 (0.32) | 2.40 (0.25) |
| 4 | 2.67 (0.58) | 2.82 (0.35) | 3.10 (0.14) |
| 6 | 3.98 (0.98) | 3.80 (0.48) | 4.74 (0.24) |
| 7 | 4.71 (0.91) ^a^ | 4.80 (0.54) ^a^ | 6.61 (0.84) |

Data are expressed as relative value to initial tumor volume mean (SD).

Significance was determined by one-way analysis of variance followed by Tukey-Kramer post hoc test.

^a^*p* < 0.05 vs vehicle.

**
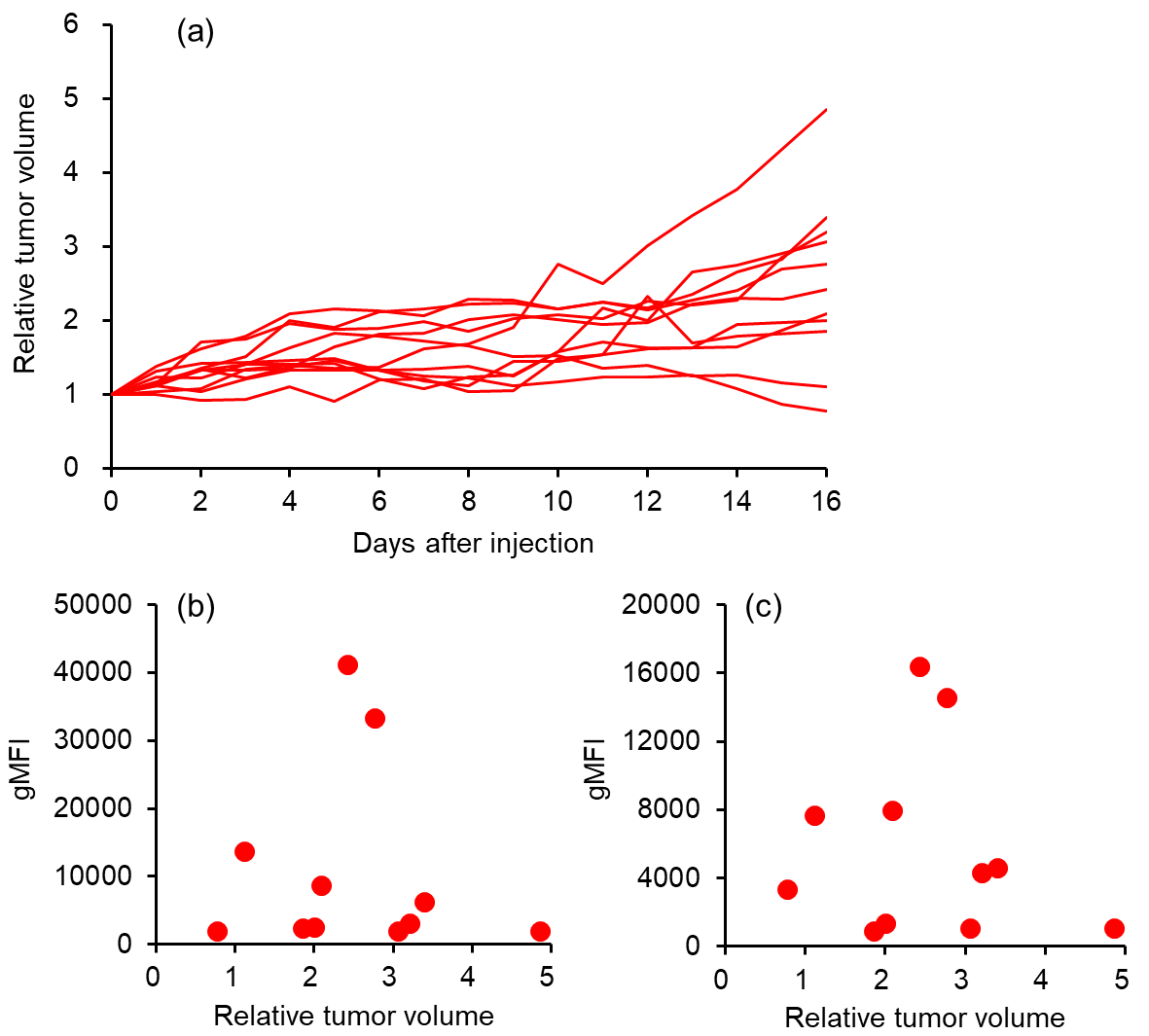
**

**Figure S3.** (a) Relative tumor volume of Colon-26 tumor-bearing BALB/c mice treated with [^211^At]**1** (675 kBq) + αPD-1 used for flow cytometry. (b) Individual relative tumor volume and geometric mean fluorescence intensity (gMFI) of CD4^+^ T cells (CD4⁺CD45⁺ live cells). (c) Individual relative tumor volume and gMFI of CD8^+^ T cells (CD8a⁺CD45⁺ live cells). Flow cytometry was performed on day 16 after treatment, when the differences in therapeutic efficacy were observed.

**Reference**

1. Echigo H, Mishiro K, Munekane M, Fuchigami T, Washiyama K, Takahashi K, et al. Development of probes for radiotheranostics with albumin binding moiety to increase the therapeutic effects of astatine-211 (^211^At). Eur J Nucl Med Mol Imaging. 2024;51:412-21. doi:10.1007/s00259-023-06457-0.
